# Boracic Acid for Diabetes

**Published:** 1886-08

**Authors:** 


**Boracic Acid for Diabetes.**—F. A. Monckton reports in the *Australian Medical Gazette* a case of diabetes mellitus cured by the use of this drug. He says, while pointing out that the value of boric acid as a diabetic remedy has only been proved in this one case, let me earnestly beg that those who have an opportunity of watching its effect will try it. When placed on the boric acid the patient's urine had a specific gravity of 1,025. Seven grains of the acid were given three times a day, and at the end of ten weeks the specific gravity was 1,016; no sugar. He continues the drug, however, as it produces no unpleasant effects. No stringent dietary regulations were observed in this case.—*Medical World.*

